# Association between treatment failure and hospitalization after receipt of neutralizing monoclonal antibody treatment for COVID-19 outpatients

**DOI:** 10.1186/s12879-022-07819-z

**Published:** 2022-11-07

**Authors:** David J. Douin, Adane F. Wogu, Laurel E. Beaty, Nichole E. Carlson, Tellen D. Bennett, Neil R. Aggarwal, David A. Mayer, Toan C. Ong, Seth Russell, Jeffrey Steele, Jennifer L. Peers, Kyle C. Molina, Matthew K. Wynia, Adit A. Ginde

**Affiliations:** 1 Department of Anesthesiology, University of Colorado School of Medicine, 12401 E. 17th Avenue, B-215, Aurora, CO 80045 USA; 2 Department of Biostatistics and Informatics, Colorado School of Public Health, Aurora, CO USA; 3 Colorado Clinical and Translational Sciences Institute, University of Colorado Anschutz Medical Campus, Aurora, CO USA; 4 Section of Informatics and Data Science, Department of Pediatrics, University of Colorado School of Medicine, Aurora, CO USA; 5 Department of Medicine, University of Colorado School of Medicine, Aurora, CO USA; 6 Research Informatics, Children’s Hospital Colorado, Aurora, CO USA; 7 Department of Emergency Medicine, University of Colorado School of Medicine, Aurora, CO USA; 8 Division of Infectious Diseases, University of Colorado School of Medicine, Aurora, CO USA; 9 University of Colorado School of Pharmacy and Pharmaceutical Sciences, Aurora, CO USA; 10 Section of General Internal Medicine, Department of Medicine, University of Colorado School of Medicine, Aurora, CO USA

**Keywords:** COVID-19, SARS-CoV-2, Monoclonal antibodies

## Abstract

**Background:**

Neutralizing monoclonal antibodies (mAbs) are highly effective in reducing hospitalization and mortality among early symptomatic COVID-19 patients in clinical trials and real-world data. While resistance to some mAbs has since emerged among new variants, characteristics associated with treatment failure of mAbs remain unknown.

**Methods:**

This multicenter, observational cohort study included patients with COVID-19 who received mAb treatment between November 20, 2020, and December 9, 2021. We utilized electronic health records from a statewide health system plus state-level vaccine and mortality data. The primary outcome was mAb treatment failure, defined as hospitalization or death within 28 days of a positive SARS-CoV-2 test.

**Results:**

COVID-19 mAb was administered to 7406 patients. Hospitalization within 28 days of positive SARS-CoV-2 test occurred in 258 (3.5%) of all patients who received mAb treatment. Ten patients (0.1%) died within 28 days, and all but one were hospitalized prior to death. Characteristics associated with treatment failure included having two or more comorbidities excluding obesity and immunocompromised status (adjusted odds ratio [OR] 3.71, 95% confidence interval [CI] 2.52–5.56), lack of SARS-CoV-2 vaccination (OR 2.73, 95% CI 2.01–3.77), non-Hispanic black race/ethnicity (OR 2.21, 95% CI 1.20–3.82), obesity (OR 1.79, 95% CI 1.36–2.34), one comorbidity (OR 1.68, 95% CI 1.11–2.57), age ≥ 65 years (OR 1.62, 95% CI 1.13–2.35), and male sex (OR 1.56, 95% CI 1.21–2.02). Immunocompromised status (none, mild, or moderate/severe), pandemic phase, and type of mAb received were not associated with treatment failure (all p > 0.05).

**Conclusions:**

Comorbidities, lack of prior SARS-CoV-2 vaccination, non-Hispanic black race/ethnicity, obesity, age ≥ 65 years, and male sex are associated with treatment failure of mAbs.

**Supplementary Information:**

The online version contains supplementary material available at 10.1186/s12879-022-07819-z.

## Introduction

Persistent surges of coronavirus disease 2019 (COVID-19) necessitate novel therapeutics, especially for unvaccinated persons, those with waning vaccine immunity, or older adults with chronic medical conditions [1]. Neutralizing monoclonal antibodies (mAbs) are widely seen as an important tool for managing surge caseloads. They provide immediate, passive immunity against severe acute respiratory syndrome coronavirus-2 (SARS-CoV-2), the virus that causes COVID-19. Phase III clinical trials [2–5] and real-world data [6–8] demonstrated the effectiveness of neutralizing mAbs in reducing hospitalization and mortality among early symptomatic COVID-19 patients. Based on the strength of these trials, the US Food and Drug Administration (FDA) currently recommends mAb therapy for non-hospitalized patients when both ritonavir-boosted nirmatrelvir (Paxlovid) and remdesivir are not available, feasible to use, or clinically appropriate [9].

While some mAbs have lost effectiveness due to resistance of newer variants [10, 11], other mAb agents and antivirals have maintained effectiveness. When these agents are effective, hospitalization or death (e.g., treatment failure) occurs infrequently but are important outcome measures to understand which patients might be less likely to benefit from mAb therapies. These data could inform alternate treatment, combination therapy, or intensified follow-up. Prior studies did not observe enough adverse events to analyze the factors associated with treatment failure [4, 5, 12]. Our group established a real-world evidence platform in 2021 to assess the ongoing clinical impact of mAb therapies in high-risk outpatients with early symptomatic COVID-19. We recently reported the effectiveness of mAbs in significantly reducing hospitalization and 28-day mortality among such outpatients [6, 7]. Patient characteristics associated with treatment failure of mAbs, however, remain unknown.

Accordingly, our objective was to evaluate the characteristics associated with treatment failure among high-risk outpatients treated with mAbs during different pandemic phases, such as Alpha and Delta, and broad SARS-CoV-2 vaccination. We included patients prior to the emergence of resistance to many mAbs by newer variants such as Omicron.

## Methods

### Study oversight and data sources

We completed a secondary analysis of a multicenter observational cohort study collaborating with leaders from the University of Colorado Hospital, University of Colorado Health (UCHealth), and the Colorado Department of Public Health and Environment (CDPHE). The study was approved by the Colorado Multiple Institutional Review Board (COMIRB) with a waiver of informed consent. We previously reported methods for data collection [7]. In brief, we accessed patient data from the electronic health record (EHR; Epic, Verona, WI) of UCHealth, the largest health system in Colorado. UCHealth consists of 13 hospitals across the state and accounts for approximately 141,000 annual hospital admissions. Data from the EHR were merged with statewide data on vaccination status from the Colorado Comprehensive Immunization Information System and mortality from Colorado Vital Records.

### Patient population studied

**Fig. 1:**
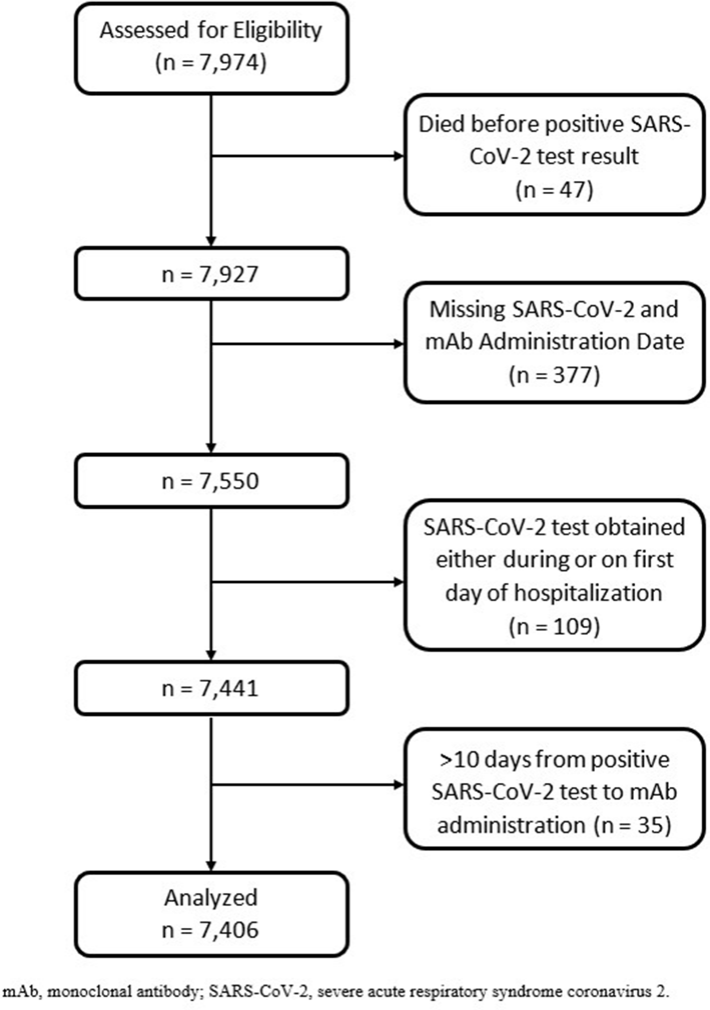
CONSORT flow chart detailing patient enrollment allocation, follow-up, and analysis

We included adult patients (≥ 18 years of age) diagnosed with SARS-CoV-2 between November 20, 2020, and December 9, 2021, who received mAb treatment within 10 days of a positive SARS-CoV-2 test (n = 7974). We identified patients using EHR-based date of SARS-CoV-2 positive testing (by polymerase chain reaction or antigen tests) or date of administration of mAb treatment (if no SARS-CoV-2 test result date was available). The decision to utilize mAb treatment was made by patients and clinicians. The CDPHE established a statewide referral system to facilitate patient referrals to facilities for mAb infusion [13]. We excluded patients who died before their SARS-CoV-2 test results, patients missing both a mAb administration date and a SARS-CoV-2 positive date, and patients who tested positive either while in the hospital or on the same day as admission as these patients were not eligible to receive mAbs and not at risk for the primary study outcome. We also excluded patients with a positive SARS-CoV-2 test after December 9, 2021, to avoid comparing patients infected with the Delta variant with those infected with the Omicron variant, which had a much lower rate of hospitalization or death [14]. We also excluded patients who had more than 10 days between their SARS-CoV-2 positive test and the date of mAb administration, in accordance with the FDA’s Emergency Use Authorization (EUA) criteria (Fig. 1). For patients who were missing a documented SARS-CoV-2 test date, a test date was imputed based on the distribution of observed times between SARS-CoV-2 positive test to mAb administration.

### Outcomes

The primary outcome was mAb treatment failure, defined as hospitalization or death within 28 days of a positive SARS-CoV-2 test obtained from EHR data. Hospitalization was defined as an inpatient or observation encounter documented in the EHR. Death was defined as all-cause mortality whether the patient was hospitalized or not.

### Variable definitions

The presence and status of comorbid conditions (cardiovascular disease, hypertension, pulmonary disease, renal disease, diabetes) were determined using the Charlson and Elixhauser comorbidity indices [15] for EHR data and a 90-day lookback period. Immunocompromised status was further validated by manual chart reviews and was categorized as “Not Immunocompromised,” “Mild,” or “Moderate/Severe,” based on chronic medications or specific conditions. Mild criteria included the administration of tumor necrosis factor-alpha inhibitors, or azathioprine, as well as human immunodeficiency virus (HIV) without acquired immunodeficiency syndrome (AIDS). Moderate/Severe patients were NIH tier 1 immunocompromised individuals, including those receiving chemotherapeutic agents or antirejection medications, and HIV with AIDS (Additional file 5: Table S1). If a patient met at least one defining element for “Moderate/Severe,” they were categorized as “Moderate/Severe” immunocompromised. Systemic corticosteroids, excluding dexamethasone, were classified as “Mild” immunocompromised. The number of comorbid conditions was created by summing all conditions excluding immunocompromised status or obesity because these two were the key pre-specified conditions we chose to evaluate. Pandemic phase was divided based on SARS-CoV-2 positive date and in accordance with the prevalent variant in Colorado. Phases included Pre-Alpha (November 2020–February 2021), Alpha (March 2021–June 2021), and Delta (June 2021–December 2021). Virus sequencing results were not available at an individual patient level. Vaccination status at the time of SARS-CoV-2 positive date was categorized by the number of vaccine doses received (zero, one, two, or more) at least 2 weeks before infection. mAb treatments included bamlanivimab (Eli Lilly), casirivimab + imdevimab (Regeneron), bamlanivimab + etesevimab (Eli Lilly), and sotrovimab (GlaxoSmithKline).

### Statistical analysis

Patient characteristics by treatment failure status were compared using t-tests and Chi-squared test statistics, as appropriate. Due to the infrequent nature of our primary outcome, Firth’s bias-reduced multiple logistic regression model was used to investigate the association between patient risk factors and treatment failure [16]. For each of the risk factors in the model, we computed the adjusted odds ratio (OR) and 95% confidence intervals (95% CI) via penalized profile likelihood. A separate model was fitted for each risk factor along with a final multivariable model including age, sex, race/ethnicity, insurance status, presence of obesity, insurance status, immunocompromised status, number of comorbidities, pandemic phase, and vaccination status to adjust association estimates for potential confounding effects between risk factors. In addition, we computed the absolute risk difference along with 95% CI. Kaplan–Meier curves were estimated to visually assess temporal trends in cumulative incidence by treatment status for secondary outcomes.

We performed two sensitivity analyses. For the first, we employed a more conservative imputation method for missing SARS-CoV-2 positive test dates by assuming all missing positive test dates were 10 days prior to the mAb administration date (the maximum time difference allowed by the EUA). Second, we included only patients with complete dates for both SARS-CoV-2 positive test and mAb administration dates verified by EHR data. All statistical analyses were performed using R Statistical Software (version 3.6.0; R Foundation for Statistical Computing, Vienna, Austria).

## Results

### Treatment failure of mAbs

A total of 7406 patients with confirmed SARS-CoV-2 infection received mAbs between November 20, 2020, and December 9, 2021. A CONSORT flow diagram of applied exclusion criteria is presented in Fig. 1. We excluded 47 patients who died before their SARS-CoV-2 test results, 377 patients missing both a mAb administration date and a SARS-CoV-2 positive date, and 109 patients who tested positive for SARS-CoV-2 either while in the hospital or on the same day as admission. We also excluded 35 patients who had more than 10 days between their SARS-CoV-2 positive test and the date of mAb administration, in accordance with EUA criteria. Treatment failure within 28 days of a positive SARS-CoV-2 test occurred in 258 (3.5%) patients who received mAbs. Ten patients (0.1%) died within 28 days of their positive test, and all but one of these patients were hospitalized prior to death. The maximum level of oxygenation support required during the index hospitalization for the 258 patients who experienced treatment failure is displayed in Additional file 1: Figure S1.

### Characteristics of hospitalized and not-hospitalized cohorts

Patients who experienced treatment failure were older (47% vs. 33% were age ≥ 65 years), more likely to be male (55% vs. 44%), more likely to be obese (46% vs. 27% with body mass index [BMI] ≥ 30 kg/m^2^), more likely to be at least mildly immunocompromised (37% vs. 23%) and more likely to have two or more additional comorbidities (63% vs. 30%) (Table 1).

**Table 1:** Characteristics of COVID-19 patients receiving outpatient mAb treatment, stratified by hospitalization status

Characteristics, n (%)	No treatment failure n = 7148 (96.5%)	Treatment failure n = 258 (3.5%)	p-value
Age in years			< 0.001
18–44	2055 (28.7)	54 (21.0)	
45–64	2767 (38.7)	84 (32.3)	
≥ 65	2326 (32.5)	120 (46.7)	
Sex			< 0.001
Male	3172 (44.4)	142 (55.3)	
Female	3976 (55.6)	116 (44.7)	
Race/ethnicity			0.01
Non-Hispanic white	5596 (81.3)	194 (75.5)	
Non-Hispanic black	178 (2.6)	14 (5.4)	
Hispanic	760 (11.0)	37 (14.4)	
Other	351 (5.1)	12 (4.7)	
Insurance status			< 0.001
Private/commercial	3931 (55.0)	94 (36.4)	
Medicare	2292 (32.1)	124 (48.1)	
Medicaid	511 (7.1)	32 (12.4)	
None/uninsured	255 (3.6)	3 (1.2)	
Other/unknown	159 (2.2)	5 (1.9)	
Obesity*	1896 (27.1)	119 (46.1)	< 0.001
Number of comorbidities			< 0.001
0	2825 (40.4)	39 (15.1)	
1	2042 (29.2)	56 (21.7)	
≥ 2	2129 (30.4)	163 (63.2)	
Cardiovascular disease	1166 (16.7)	112 (43.4)	< 0.001
Hypertension	2743 (39.2)	169 (65.5)	< 0.001
Pulmonary disease	2022 (28.9)	99 (38.5)	0.001
Renal disease	618 (8.8)	77 (29.8)	< 0.001
Diabetes	1136 (15.9)	89 (34.5)	< 0.001
Immunocompromised			< 0.001
None	5543 (77.5)	163 (63.2)	
Mild	793 (11.1)	38 (14.7)	
Moderate/severe	812 (11.4)	57 (22.1)	
SARS-CoV-2 vaccine doses			< 0.001
2+	2767 (38.7)	61 (23.6)	
1	503 (7.0)	16 (6.2)	
0	3878 (54.3)	181 (70.2)	
Pandemic phase			< 0.001
Pre-Alpha	372 (5.2)	25 (9.7)	
Alpha	422 (5.9)	30 (11.6)	
Delta	6354 (88.9)	203 (78.7)	
Days to mAb treatment			0.33
1 or less	1604 (22.4)	70 (27.1)	
2	1396 (19.5)	50 (19.4)	
3	1319 (18.5)	48 (18.6)	
4	1041 (14.6)	36 (14.0)	
5	807 (11.3)	31 (12.0)	
6	456 (6.4)	11 (4.3)	
7–10	525 (7.3)	12 (4.7)	
mAb type			0.008
Bamlanivimab	400 (5.6)	24 (9.3)	
Bamlanivimab + etesevimab	1003 (14.1)	30 (11.6)	
Bamlanivimab + imdevimab	5190 (72.6)	194 (75.2)	
Sotrovimab	541 (7.6)	10 (3.9)	

*mAb* monoclonal antibody, *SARS-CoV-2* severe acute respiratory syndrome coronavirus 2

BMI data were missing from a total of 2696 patients (36%). Of these patients, 2691 (38%) were from the no-treatment failure group, and 5 (2%) from the treatment failure group

### Primary analysis

**Fig. 2:**
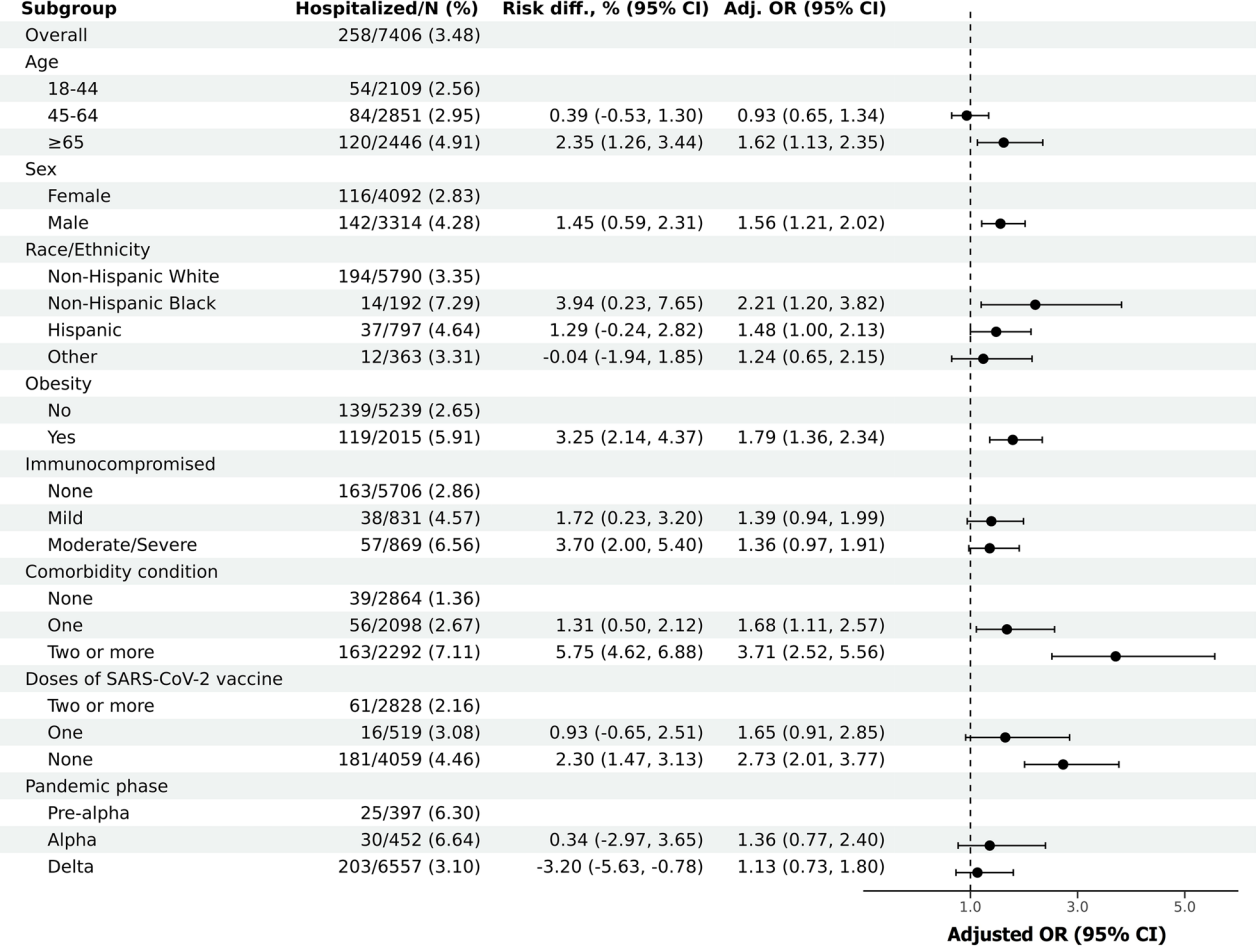
Adjusted risk difference and adjusted odds ratio (OR) for treatment failure for each risk factor from the full model. Risk differences were calculated via Firth’s bias-reduced multiple regression logistic regression. Adjusted ORs and 95% confidence intervals (95% CI) were computed by penalized profile likelihood

All patient risk factors were significantly associated with treatment failure during initial (unadjusted) analysis (Table 1). After accounting for potential confounding of insurance status and different comorbid conditions, the following risk factors remained as significant independent predictors of treatment failure: age ≥ 65 years old compared to those younger than 45 years old (adjusted OR 1.62, 95% CI 1.13–2.35, p = 0.008), male versus female sex (OR 1.56, 95% CI 1.21–2.02, p = 0.001), Non-Hispanic black race versus non-Hispanic white race (OR 2.21, 95% CI 1.20–3.82, p = 0.013), and obesity (OR 1.79, 95% CI 1.36–2.34, p < 0.001) (Fig. 2). The cumulative number of additional comorbid conditions was also significantly associated with hospitalization: patients with one additional comorbidity had OR 1.68 (95% CI 1.11–2.57, p = 0.014), and those with two or more additional comorbidities had OR 3.71 (95% CI 2.52–5.56, p < 0.001) compared to those with no additional comorbidities. Compared to patients who received two or more doses of vaccines, those who received no SARS-CoV-2 vaccine had a nearly three times higher likelihood of experiencing treatment failure (adjusted OR 2.73, 95% CI 2.01–3.77, p < 0.001).

**Fig. 3:**
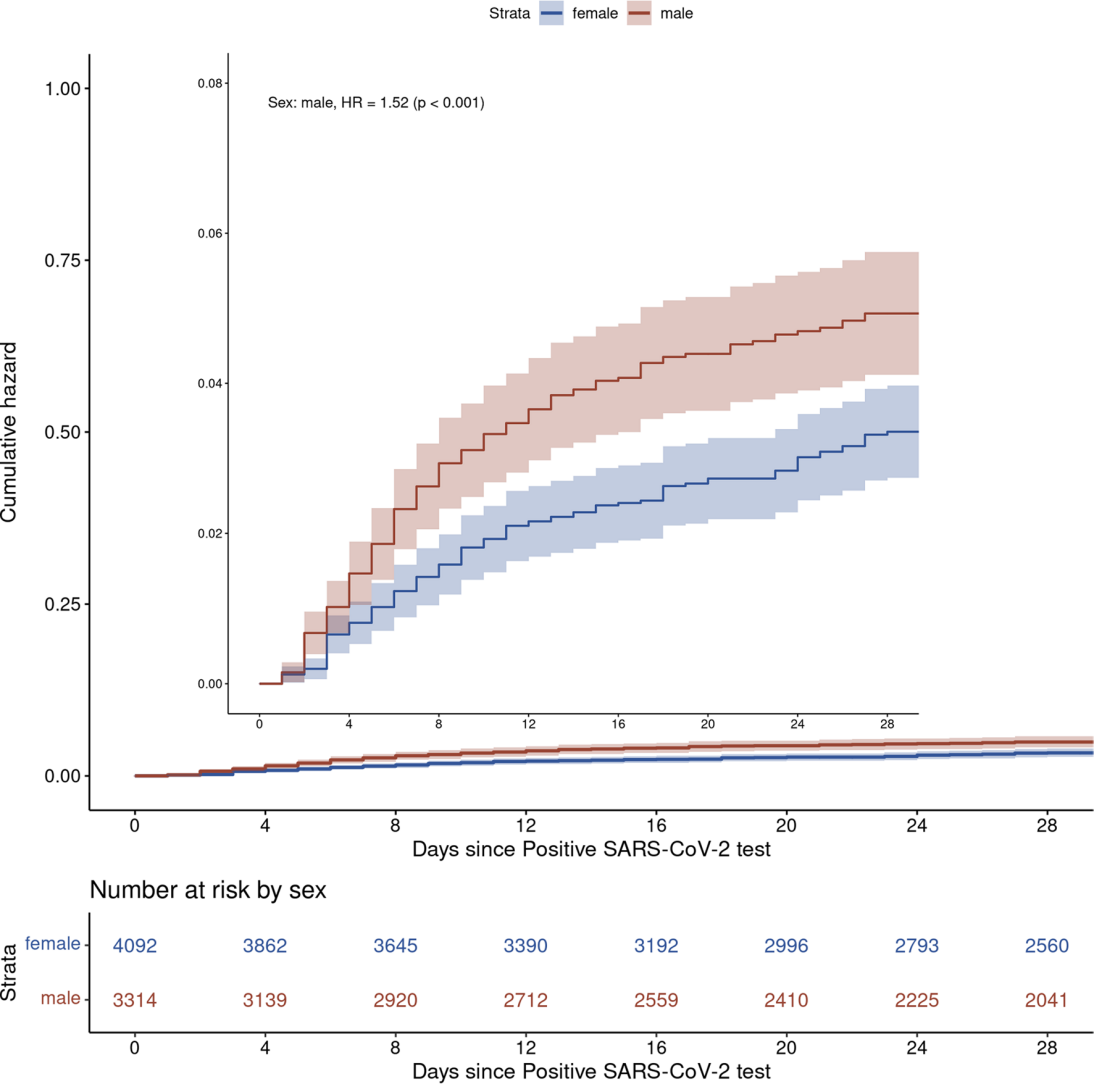
Cumulative incidence of hazard for hospitalization by sex

**Fig. 4:**
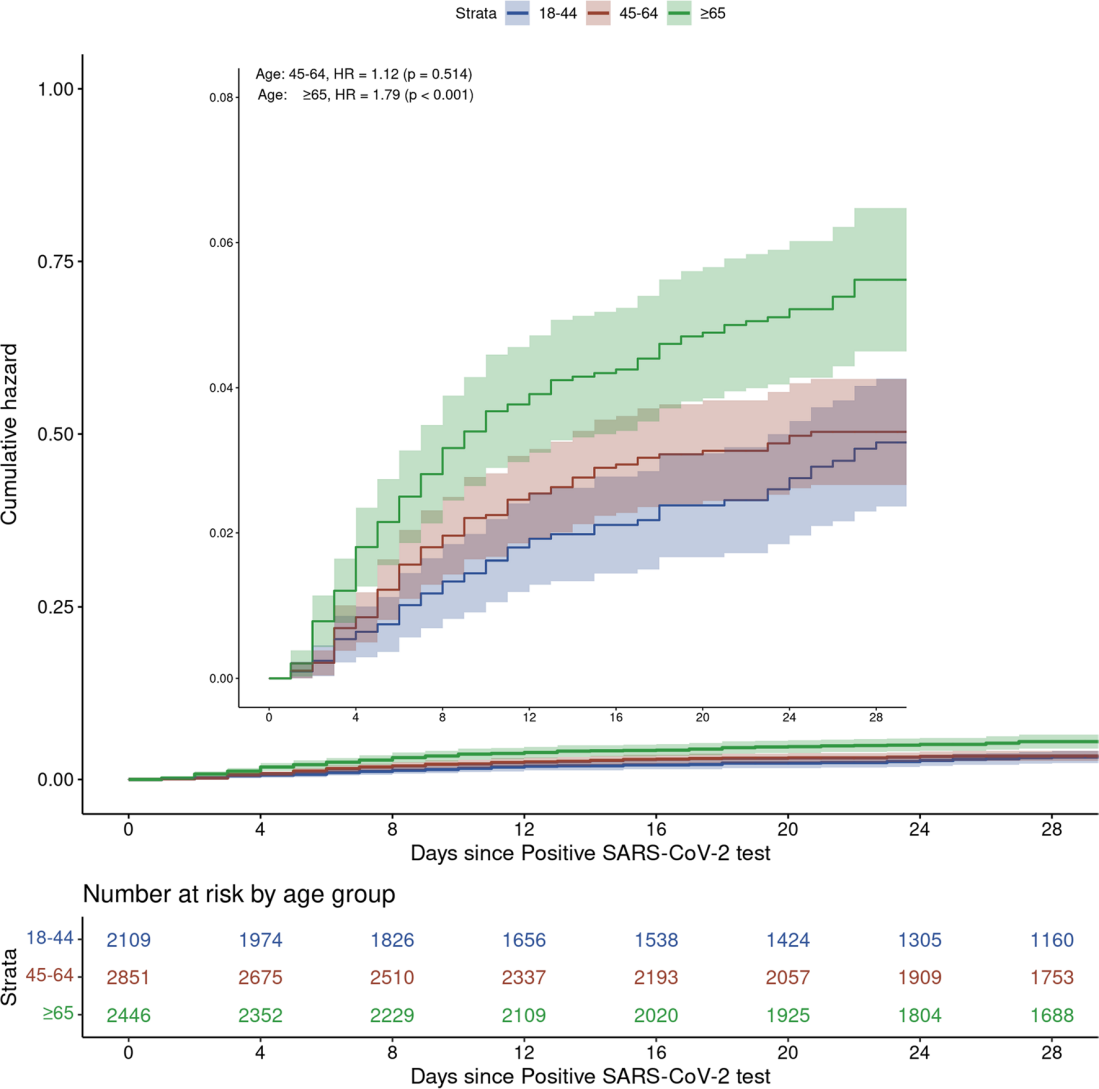
Cumulative incidence of hazard for hospitalization by age-group

Presence of at least mild immunocompromised status was significantly more likely in hospitalized patients compared to non-hospitalized patients in univariate analysis (37% vs. 23% in Table 1; unadjusted OR 2.02, 95% CI 1.55–2.61, p < 0.001). However, after adjusting for other risk factors, neither mild immunocompromised status (OR 1.39, 95% CI 0.94–1.99) nor moderate/severe immunocompromised status (OR 1.36, 95% CI 0.97–1.91) was significantly associated with treatment failure, compared to non-immunocompromised patients (Fig. 2). Pandemic phase and type of mAb received were also not associated with treatment failure. The cumulative incidence of hospitalization stratified by immunocompromised status is displayed in Additional file 2: Figure S2, by sex in Fig. 3, and by patient age in Fig. 4. After adjusting for relevant characteristics, increasing age was significantly associated with treatment failure; however, age 45–64 years was not (Fig. 2).

### Sensitivity analyses

We first employed a more conservative imputation method for missing SARS-CoV-2 positive test dates by assuming all missing positive test dates were 10 days prior to the mAb administration date (the maximum time difference allowed by the EUA). In that sensitivity analysis, we found that Hispanic ethnicity (adjusted OR 1.63, 95% CI 1.13–2.31, p = 0.01) and moderate/severe immunocompromised status (OR 1.42, 95% CI 1.02–1.97; p = 0.04) were significantly associated with hospitalization (Additional file 3: Figure S3). We did not observe any other differences between our primary analysis and this analysis for other risk factors. In this analysis, the overall sample size changed slightly because SARS-CoV-2 positive test date was used in defining our initial eligibility criteria. For the second sensitivity analysis, we included only patients with confirmed dates for both SARS-CoV-2 positive test and mAb administration. In this cohort, we found that age ≥ 65 years was not associated with treatment failure (OR 1.65, 95% CI 0.98–2.82), and patients who received only one dose of SARS-CoV-2 vaccine had a higher risk of experiencing treatment failure compared to those who had two or more doses of vaccine (OR 2.55, 95% CI 1.19–5.16, p = 0.02) (Additional file 4: Figure S4). No other differences were observed between this analysis and our primary analysis.

## Discussion

Our study, using real-world data in the pre-Omicron variant period, demonstrates novel results that age ≥ 65 years, male sex, non-Hispanic black race/ethnicity, obesity, increasing number of comorbidities, and lack of prior SARS-CoV-2 vaccination are significantly associated with treatment failure (hospitalization or death) following treatment of COVID-19 with mAbs. While some neutralizing mAbs have lost effectiveness to Omicron and its sub-variants, mAbs that have maintained effectiveness are widely regarded as critical tools in combating surges in COVID-19 cases. However, previous studies have not evaluated the patient characteristics associated with treatment failure of mAbs. Our present study fills this key knowledge gap.

Hospitalization among all patients who received mAbs was uncommon (3.5%), as was 28-day mortality (0.1%). However, these patients represent an important subgroup. Identifying patients more likely to experience mAb treatment failure helps to identify those who may benefit from alternative treatment or additional follow-up. For instance, some patients may require other antiviral treatments—either as an alternative or in combination with mAb treatment. These alternative strategies could be particularly relevant during periods of hospital and ICU strain during the Covid-19 pandemic [17]. To our knowledge, our study is the first to address this critical gap in the understanding of treatment failure of mAbs. Our large sample size affords a high degree of precision in our estimates and may assist clinicians in making treatment decisions based on patient characteristics [18].

Treatment failure was progressively less likely among patients who had received more SARS-CoV-2 vaccine doses. Notably, the odds of 28-day hospitalization among mAb-treated patients who received two (or more) vaccine doses was approximately one-third that of patients who received zero doses (i.e., unvaccinated). These data support the policy that SARS-CoV-2 vaccination should remain the first-line intervention to prevent Covid-19 hospitalization. Treatment with mAbs should therefore be reserved as supplemental therapy for breakthrough COVID-19 in the high-risk patients highlighted in our study.

Interestingly, immunocompromised status was not significantly associated with treatment failure. The presence of immunocompromising conditions has previously been associated with worsened disease severity among Covid-19 patients [19–21]. Univariate analysis did reveal a strong association between at least mild immunocompromised status and treatment failure (OR 2.02, 95% CI 1.55–2.61). One of two sensitivity analyses demonstrated an association between moderate/severe immunocompromised status and treatment failure (OR 1.42, 95% CI 1.02–1.97) (Additional file 3: Figure S3). However, there appears to be collinearity between an immunocompromised status and other comorbidities, most notably cardiovascular disease (OR 12.98, 95% CI 11.31–14.91). This relationship could make sense clinically as patients with a compromised immune system may be more likely to have co-existing disease processes. However, understanding the etiology of this relationship is beyond the scope of our study. Notably, our pre-specified definition of comorbidities did not include immunocompromised status or obesity because these were key pre-specified subgroups to evaluate separately.

## Limitations

Our study has several potential limitations. We analyzed patients from a single health care system. While this large system encompasses both urban and rural settings, and community and academic hospitals, it is located entirely within one US state. Ethnic and racial minority representation was lower than national averages, which potentially limited our ability to detect differences across these subgroups. Duration of immunocompromised status was not captured; therefore, contrasting timing or duration of immunocompromising conditions with regard to treatment failure was not possible. Since our study was observational, associations may have been influenced by unmeasured confounding. Data for mortality and vaccination status were collected using a statewide database, but hospitalizations were only collected within the single health care system. Therefore, discrepancies between mAb-treated patients and hospitalization status (i.e., patients who were hospitalized outside of the health care system) may exist. In addition, death was rare, and only one additional patient died without evidence of hospital admission. However, the composite approach to treatment failure—including both hospitalization and death—is most clinically relevant. Clinical indication for hospital admission was not captured. Therefore, it is possible some of the hospitalized patients were admitted for non-COVID-19 indications. Finally, we included patients prior to the emergence of the Omicron variant. This variant has proven resistant to treatment with many mAbs [22, 23]. Patients infected with the Omicron variant may have a greater likelihood of experiencing treatment failure for some mAb and antiviral agents.

## Conclusion

Using real-world data, we found that age ≥ 65 years, male sex, non-Hispanic black race/ethnicity, increasing number of comorbidities, and lack of prior SARS-CoV-2 vaccination are significantly associated with treatment failure and hospitalization following treatment with mAbs. Understanding factors associated with treatment failure of mAbs allows for identification of patients who may require additional treatments and/or close follow-up.

## Supplementary Information

**Additional file 1: Figure S1.** Maximum level of oxygenation support during the index hospitalization for patients who experienced treatment failure. IV: invasive mechanical ventilation; HFC: high flow nasal cannula; NIV: non-invasive ventilation.

**Additional file 2: Figure S2.** Cumulative incidence of hazard for hospitalization by immunocompromised status.

**Additional file 3: Figure S3.** Adjusted risk difference and adjusted odds ratio (OR) for treatment failure for each risk factor from a conservative imputation model. In this model, we assumed all missing SARS-CoV-2 positive test dates were ten days prior to the mAb administration date. Risk differences were calculated via Firth's bias-reduced multiple regression logistic regression. Adjusted ORs and 95% confidence intervals (95% CI) were computed by penalized profile likelihood.

**Additional file 4: Figure S4.** Adjusted risk difference and adjusted odds ratio (OR) for treatment failure for each risk factor from a conservative imputation model. In this model, we included only patients with confirmed dates for both SARS-CoV-2 positive test and mAb administration. Risk differences were calculated via Firth's bias-reduced multiple regression logistic regression. Adjusted ORs and 95% confidence intervals (95% CI) were computed by penalized profile likelihood.

**Additional file 5: Table S1. **Medications and conditions used to stratify Mild versus Moderate/Severe immunocompromised status.

## Data Availability

We accessed patient data from the electronic health record (EHR; Epic, Verona, WI) of the University of Colorado Health (UCHealth), the largest health system in Colorado. UCHealth consists of 13 hospitals across the state and accounts for approximately 141,000 annual hospital admissions. Data from the EHR were merged with statewide data on vaccination status from the Colorado Comprehensive Immunization Information System and mortality from Colorado Vital Records. The data that support the findings of this study are available from Health Data Compass Warehouse Project (healthdatacompass.org), but restrictions apply to the availability of these data, which were used under license for the current study, and so are not publicly available. Data are, however, available from the authors upon reasonable request and with permission of the Health Data Compass Warehouse Project (healthdatacompass.org).
