# Supplementary figures and images for: Association between treatment failure and hospitalization after receipt of neutralizing monoclonal antibody treatment for COVID-19 outpatients
Source: BMC Infect Dis. 2022 Nov 7;22:818. doi: 10.1186/s12879-022-07819-z (PMC9639288; doi:10.1186/s12879-022-07819-z)

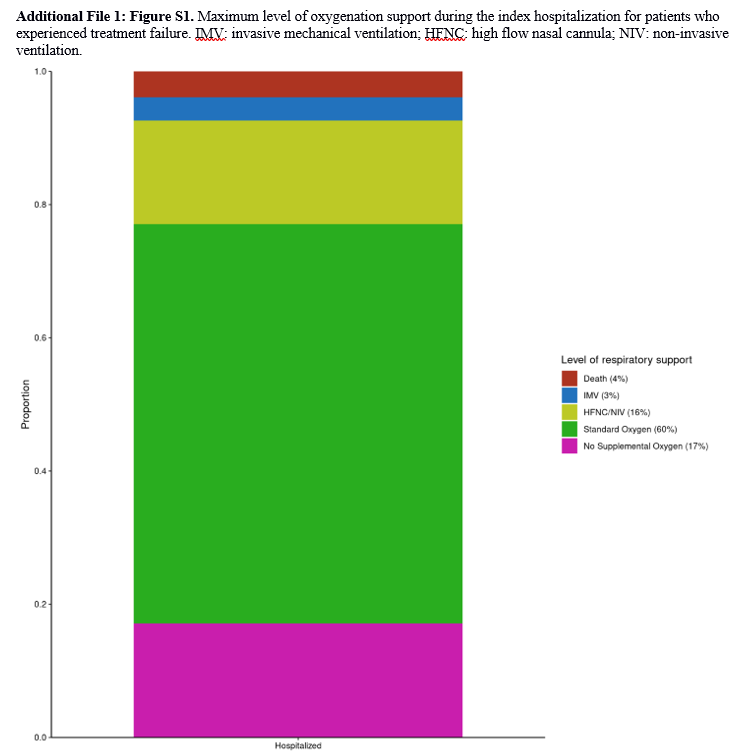

Supplement: Supplementary file 1 — Additional file 1: Figure S1. Maximum level of oxygenation support during the index hospitalization for patients who experienced treatment failure. IV: invasive mechanical ventilation; HFC: high flow nasal cannula; NIV: non-invasive ventilation. [file 12879_2022_7819_MOESM1_ESM.png]

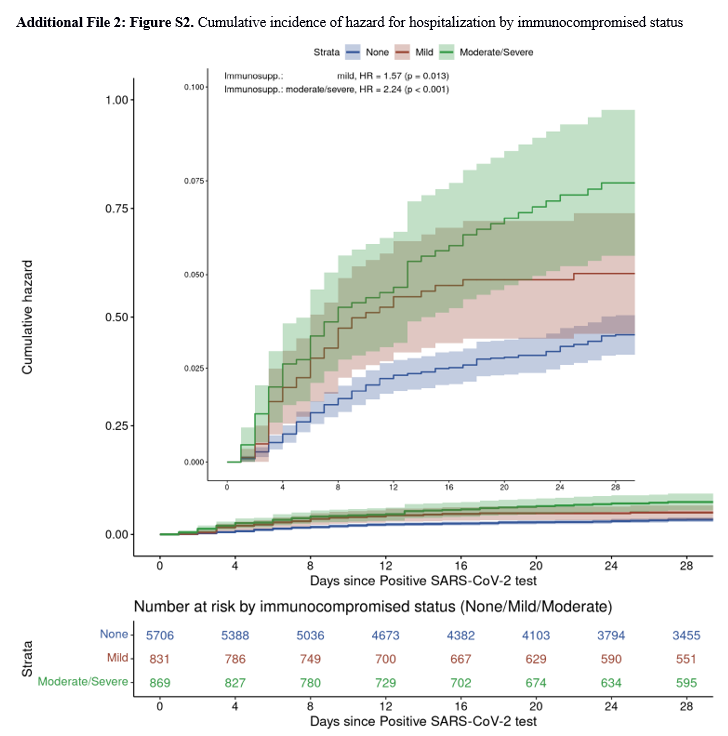

Supplement: Supplementary file 2 — Additional file 2: Figure S2. Cumulative incidence of hazard for hospitalization by immunocompromised status. [file 12879_2022_7819_MOESM2_ESM.png]

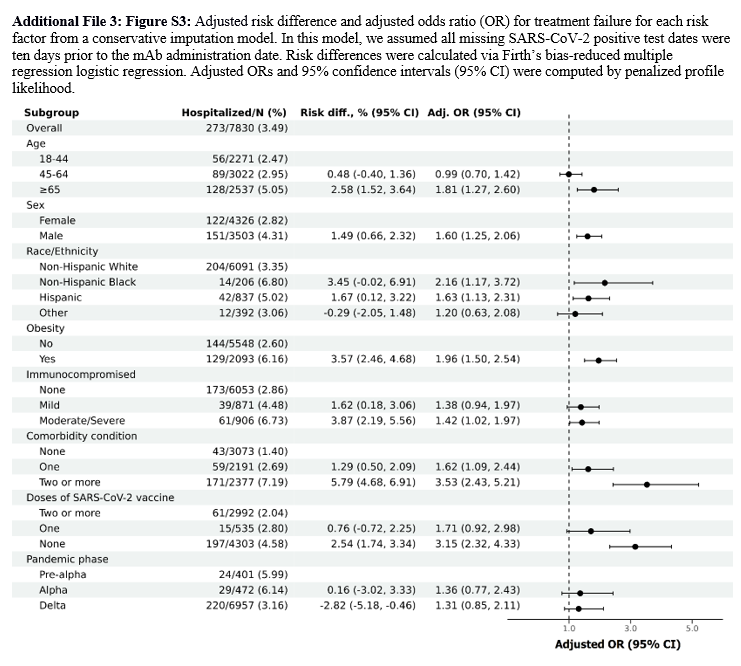

Supplement: Supplementary file 3 — Additional file 3: Figure S3. Adjusted risk difference and adjusted odds ratio (OR) for treatment failure for each risk factor from a conservative imputation model. In this model, we assumed all missing SARS-CoV-2 positive test dates were ten days prior to the mAb administration date. Risk differences were calculated via Firth's bias-reduced multiple regression logistic regression. Adjusted ORs and 95% confidence intervals (95% CI) were computed by penalized profile likelihood. [file 12879_2022_7819_MOESM3_ESM.png]

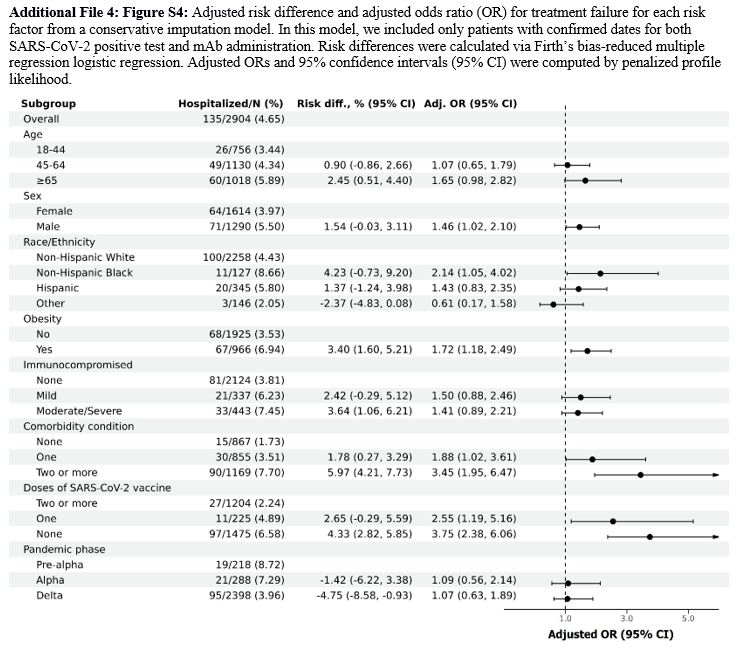

Supplement: Supplementary file 4 — Additional file 4: Figure S4. Adjusted risk difference and adjusted odds ratio (OR) for treatment failure for each risk factor from a conservative imputation model. In this model, we included only patients with confirmed dates for both SARS-CoV-2 positive test and mAb administration. Risk differences were calculated via Firth's bias-reduced multiple regression logistic regression. Adjusted ORs and 95% confidence intervals (95% CI) were computed by penalized profile likelihood. [file 12879_2022_7819_MOESM4_ESM.png]
