# Supplementary material for: Association between treatment failure and hospitalization after receipt of neutralizing monoclonal antibody treatment for COVID-19 outpatients
Source: BMC Infect Dis. 2022 Nov 7;22:818. doi: 10.1186/s12879-022-07819-z (PMC9639288; doi:10.1186/s12879-022-07819-z)
Supplement: Supplementary file 5 — Additional file 5: Table S1. Medications and conditions used to stratify Mild versus Moderate/Severe immunocompromised status. [file 12879_2022_7819_MOESM5_ESM.docx]

**Additional File 5: Table S1.** Medications and conditions used to stratify Mild versus Moderate/Severe immunocompromised status.

| **Category** | **Medications** | **Conditions** |
| --- | --- | --- |
| Not Immunocompromised | No qualifying medications | No qualifying conditions |
| Mild  (Presence of either medication or condition if moderate/severe criteria not met) | - TNF-alpha inhibitors (infliximab, etanercept, golimumab, adalimumab) - Azathioprine alone - Mycophenolate alone - Systemic Prednisone - Systemic Methylprednisolone | - Elixhauser Rheumatic - Charlson or Elixhauser HIV without mention of AIDS |
| Moderate/Severe  (Presence of either medication or condition) | - Alemtuzumab - Azathioprine plus mTORi or calcineurin inhibitor - Belatacept - Eculizumab - Rituximab - Cyclophosphamide - Mycophenolate plus mTORi or calcineurin inhibitor - Thymoglobulin - Any Calcineurin inhibitor alone - Any mTORi alone - Any Chemotherapeutic Agent - Actinomycin - Alkylating Agent - Anthracenedione - Anthracycline - Anti-Metabolite - Anti-Microtubular - Aromatase Inhibitor - CDK Inhibitor - Cytotoxic - EZH2 Inhibitor - Hedgehog Inhibitor - Immunomodulatory Imide - Immunotherapy - Microtubule Inhibitor - mTOr Kinase Inhibitor - PI3K Inhibitor - Platinum-Based - Poly (ADP-Ribose) Polymerase Inhibitor - Proteasome Inhibitor - Targeted Monoclonal Antibody - Topoisomerase Inhibitor - Tyrosine Kinase Inhibitor - Vinca Alkaloid | - Charlson or Elixhauser HIV with AIDS based on CD4 count <200 cells/μL - Lymphoma - Cancer with Metastases - Tumor |

TNF, tumor necrosis factor; mTORi, mammalian target of rapamycin; HIV, human immunodeficiency virus; AIDS, acquired immunodeficiency syndrome. CD4, cluster of differentiation 4 T-cell
